# From Persian Gulf to Indonesia: interrelated phylogeographic distance and chemistry within the genus *Peronia* (Onchidiidae, Gastropoda, Mollusca)

**DOI:** 10.1038/s41598-020-69996-8

**Published:** 2020-08-03

**Authors:** Fatemeh Maniei, Jamshid Amiri Moghaddam, Max Crüsemann, Christine Beemelmanns, Gabriele M. König, Heike Wägele

**Affiliations:** 1 Zoologisches Forschungsmuseum Alexander Koenig, Bonn, Germany; 2 Leibniz Institute for Natural Product Research and Infection Biology e.V. Hans-Knöll-Institute (HKI), Jena, Germany; 3 Institute for Pharmaceutical Biology, University of Bonn, Bonn, Germany

**Keywords:** Computational biology and bioinformatics, Classification and taxonomy, Phylogeny, Chemical biology, Chemical ecology, Metabolomics, Natural products, Networks and systems biology, Evolution, Speciation, Taxonomy

## Abstract

The knowledge of relationships between taxa is essential to understand and explain the chemical diversity of the respective groups. Here, twelve individuals of the panpulmonate slug *Peronia persiae* from two localities in Persian Gulf, and one animal of *P. verruculata* from Bangka Island, Indonesia, were analyzed in a phylogenetic and chemotaxonomic framework. Based on the ABGD test and haplotype networking using COI gene sequences of *Peronia* specimens, nine well-supported clades were found. Haplotype network analysis highlighted a considerable distance between the specimens of *P. persiae* and other clades. Metabolomic analysis of both species using tandem mass spectrometry-based GNPS molecular networking revealed a large chemical diversity within *Peronia* of different clades and localities. While *P. persiae* from different localities showed a highly similar metabolome, only few identical chemical features were found across the clades*.* The main common metabolites in both *Peronia* species were assigned as polypropionate esters of onchitriols and ilikonapyrones, and osmoprotectant amino acid-betaine compounds. On the other hand, the isoflavonoids genistein and daidzein were exclusively detected in *P. persiae*, while cholesterol and conjugated chenodeoxycholic acids were only found in *P. verruculata*. Flavonoids, bile acids, and amino acid-betaine compounds were not reported before from Onchidiidae, some are even new for panpulmonates. Our chemical analyses indicate a close chemotaxonomic relation between phylogeographically distant *Peronia* species.

## Introduction

*Peronia* Fleming, 1822, a panpulmonate slug genus, is typically distributed in tropical and subtropical regions of the Indo-Pacific ocean^1^. The genus *Peronia*, with ten acknowledged species, has been reported from many localities ranging from the Indo-Pacific to the East Coast of Africa and to Hawaii^2–4^. Due to undetected cryptic speciation, species diversity is often underestimated while the widespread distributions attributed to species is often overestimated^5^. The integration of molecular and morphological methods has led to numerous discoveries of cryptic species complexes^6^. This is also true for the genus *Peronia*, as was shown by Maniei et al.^3^.

To date, only three *Peronia* species are well investigated by molecular and/or morphology data, i.e. *P. peronii* (Cuvier, 1804), *P. verruculata* (Cuvier, 1830), and *P.* *persiae* Maniei, Espeland, Mohavedi and Wägele, 2020^2,3,6–11^. All other species are difficult to assess due to their old and superficial descriptions based on morphological characters. Additionally, many specimens that were just recently added to the National Center for Biotechnology Information (NCBI) have been characterized solely on the genus level as *Peronia* based on partial sequence data; an approach that does not allow for detailed species assignment relevant for all further investigations.

To analyze the evolutionary processes at the time of lineage separation, reconstruction of the genealogical relationship between genes can be studied by the use of phylogenetic trees or haplotype networks^12^. The phylogenetic approach assumes that the ancestral sequences are unobserved and associated with the internal nodes of the tree while the observed sequences are associated with its terminal nodes. In case observed sequences may be ancestral to each other, a network map approach might be more appropriate as these sequences will be associated with the internal nodes of the network. Therefore, haplotype network construction is a widely used approach for analyzing and visualizing the relationships among DNA sequences within a population or species^13^. Cytochrome oxidase subunit I (COI) gene sequence is a known DNA barcode to construct haplotype networks and to display the relationship among different geographical populations or species^13^.

In addition to the genomic and morphological description, chemo-taxonomy, the characterization of species-specific metabolomes, has emerged as a complementary approach in species delineation^14^. A species and its metabolome are affected by environmental factors, being exposed to abiotic (pH, temperature, pressure, oxygen, light, salinity) and biotic factors (nutrients, presence/absence of predators)^15^. A metabolomic survey will lead to valuable insights into the lifestyle of each organism and possible species-specific communication traits that might allow for predatory/prey or defensive behavior as well as intra- and interspecific communication^16^. In this context, the family Onchidiidae, living in the intertidal flats of tropical and temperate coasts encountering high fluctuation of abiotic parameters, are of special interest. So far only polypropionate esters, cholesterol, stearic acid, ethylhexylglycerins such as chimyl alcohol, and batyl alcohol have been reported from these pulmonate slugs^17–19^.

This study combines for the first time a phylogenetic and metabolomic survey of slug species belonging to the genus *Peronia*, which lives in rocky shore habitats and feeds on turf algae covering rocks^3^. In particular, the geographic relationship amongst Iranian *Peronia* haplotypes from two different localities was compared by phylogenetic reconstruction, species delimitation, and haplotype networking. Subsequently, the secretome of phylogeographical distinct species was compared to other specimens of distinct *Peronia* species widely distributed in the Indo-Pacific to identify a possible core metabolome and species-specific traits.

## Results

### Species delimitation and haplotype networking of *Peronia* species

**Figure 1:**
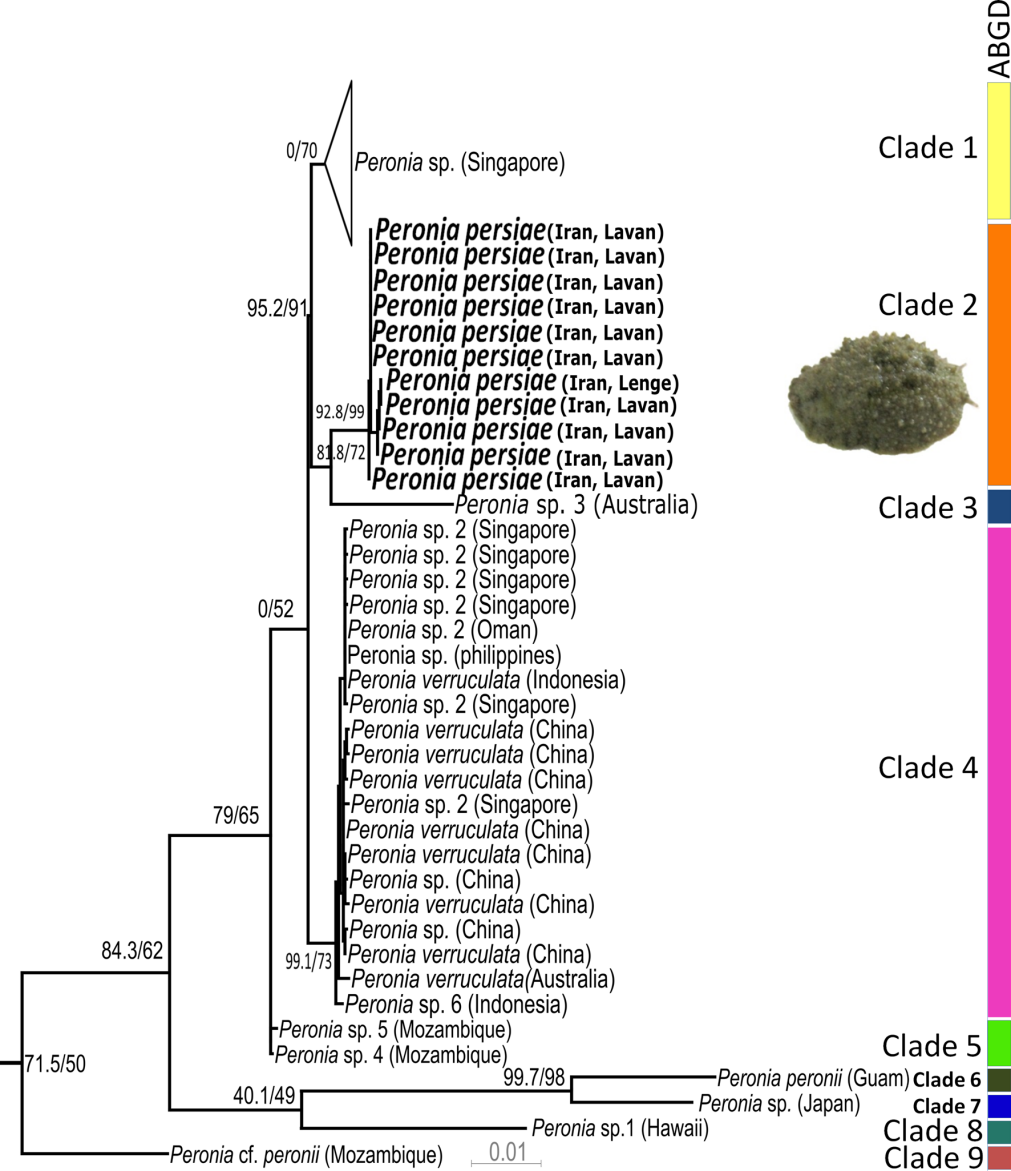
Maximum likelihood tree of the genus *Peronia* based on COI data set, *Wallaconchis graniferus* (Semper, 1880) used as outgroup (not shown here). The clades within the genus *Peronia* resulting from the ABGD test are visualized on the right side, using different colors for each clade. Numbers before and after slash indicate approximate likelihood ratio test (SH-aLRT) and ultrafast bootstrap values, respectively (graph modified from Maniei et al.^3^. Inlay picture shows one specimen of *P. persiae*.

**Figure 2:**
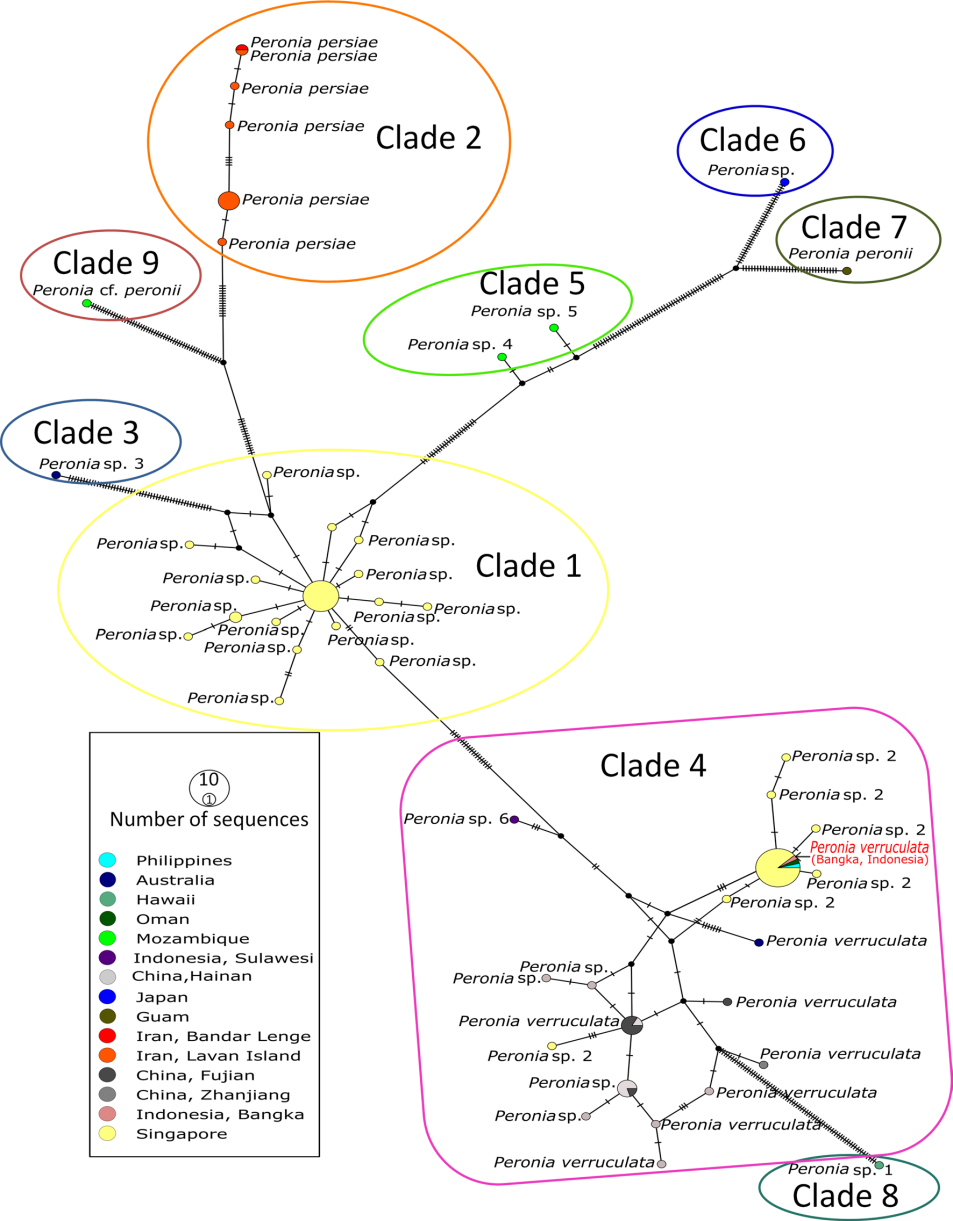
Haplotype network of all available *Peronia* COI sequences. Colored nodes on the haplotype network correspond to specific geographic localities, with each clade (see Fig. 1) highlighted by a colored box. Hash marks on the haplotype network denote mutational steps and the size of the colored nodes corresponds to the number of sequences. *P. verruculata* from Bangka, Indonesia (red text, clade 4), together with *P. persiae* specimens (clade 2) were used for molecular network analyses.

**Figure 3:**
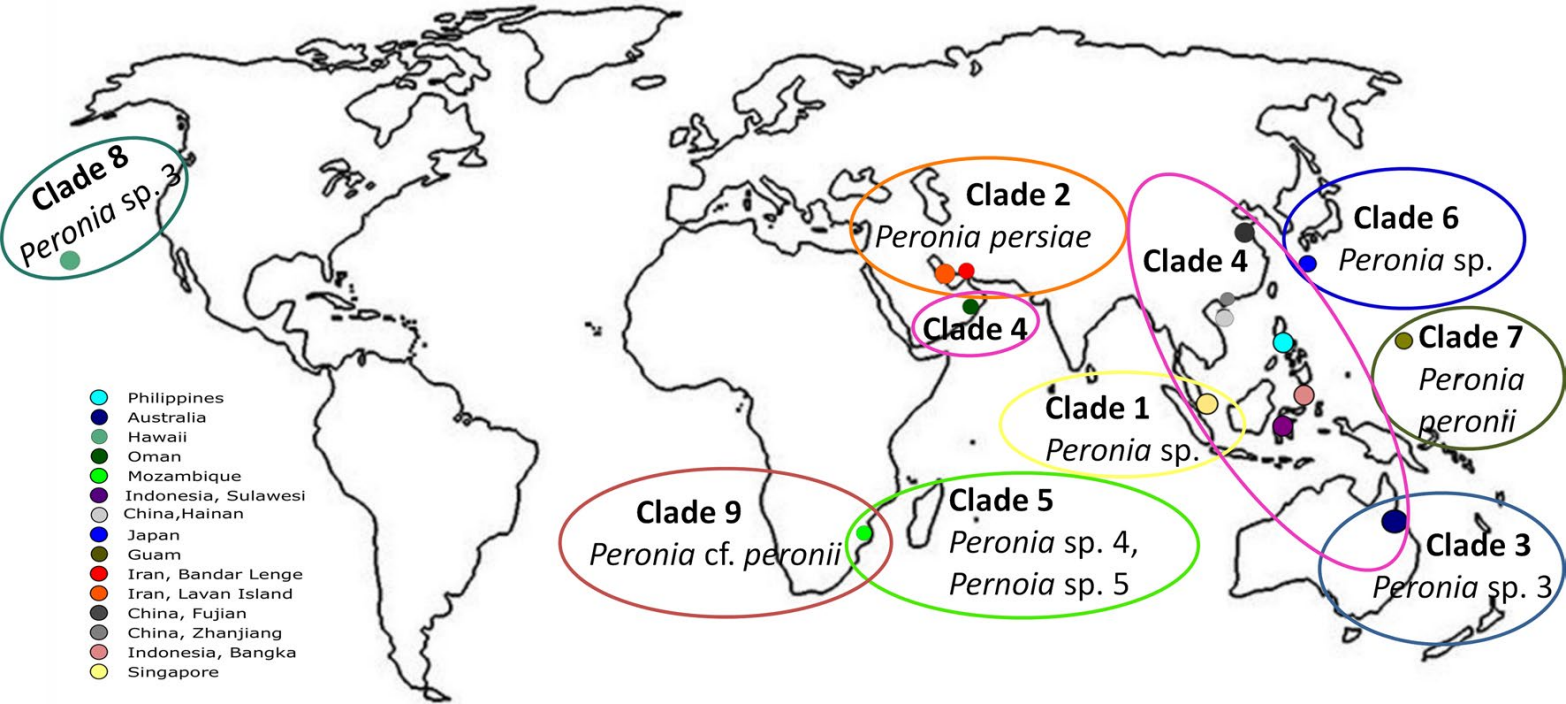
Geographical distribution map of *Peronia* in the Indo-Pacific Ocean with detailed localities and distribution of clades highlighted in the same color as in Fig. 2. The blank thick white world map—b3c is taken from outline world map images website using the following html link: https://www.outline-world-map.com/blank-thick-white-world-map-b3c.

The ABGD test results on the *Peronia* COI sequences indicated nine well-supported groups in this genus, hereafter referred to as clade 1–9 (Fig. 1). All these nine clades are also found in the haplotype network (Fig. 2) and reflect a geographical separation as shown in the distribution map of the genus (Fig. 3). The ABGD test revealed that specimens of *P. persiae* form a separate clade (clade 2). Thus, the specimens from two localities of the Persian Gulf (Iran), i.e. Bandar Lengeh and Lavan Island, were considered as a distinct new species.

According to our results, the hitherto unidentified specimen from Indonesia mentioned in Papu et al. as *Peronia* sp. a^20^ and in Maniei et al. as *P.* sp.7^3^ was placed in the clade 4, together with *P. verruculata* specimens from China and Australia. Therefore, we preliminarily assign this specimen from Indonesia to the species *P. verruculata*.

The haplotype network analysis highlights a considerable distance between the specimens of *P. persiae* and other clades, including our Indonesian haplotype, whereas only a few mutations were observed within the same species. None of the Iranian haplotypes are observed within any other clade, indicating no connectivity to any other *Peronia* populations. Interesting in this context is clade 1, which exists of specimens exclusively from Singapore, thus showing again the restricted gene flow between populations. In contrast, clade 4 (*P. verruculata*), which is also distributed in Singapore, contains haplotypes that are similar or the same as in Oman, Indonesia, China, Philippines, and even in Australia. Therefore, *P. verruculata* seems to be more widely distributed than all other clades (Fig. 3). This indicates that *P. verruculata* lives sympatric with specimens from clade 1 in Singapore, with no connectivity. The only other overlapping distribution not involving *P. verruculata* could be shown here for clade 5 with two undescribed *Peronia* specimens and a *Peronia* cf. *peronii* in Mozambique.

However, it needs to be emphasized that the number of available sequences for all other clades is very limited and further sampling is required.

### Molecular networking of *Peronia persiae* (clade 2) and *P. verruculata* (clade 4)

To evaluate species-specific differences as well as locality dependent differences, we explored the chemical features/compounds in two *Peronia* species of different clades as well as the same species from two different localities using MS/MS-based Global Natural Product Social molecular networking (GNPS)^21^.

**Figure 4:**
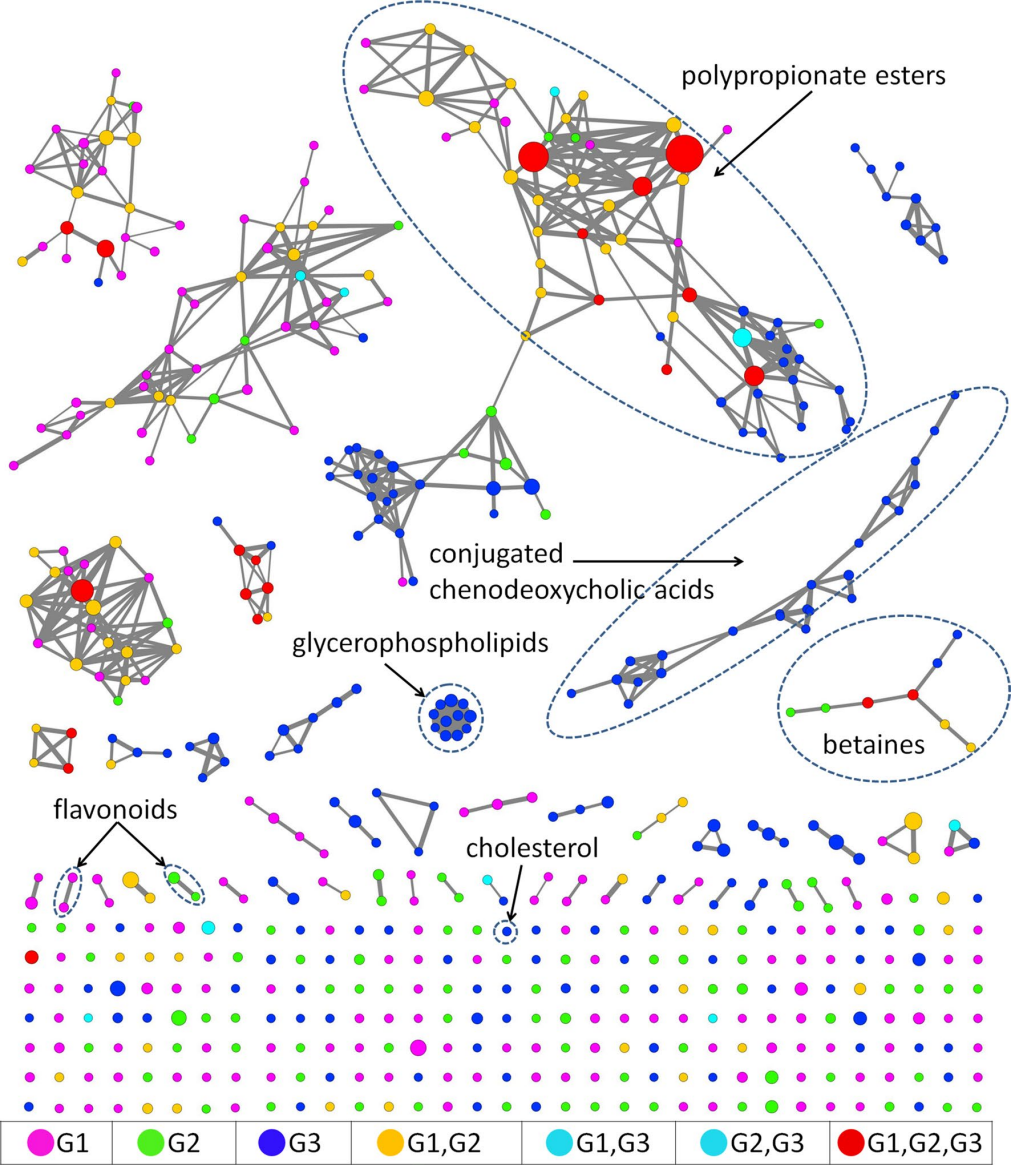
Molecular network of *Peronia persiae* specimens and one *P. verruculata* specimen. The network is color-coded according to detection from single or multiple groups. Dereplicated compounds are marked and the node size reflects the number of the spectra detected from each parent ion. G1: *P. persiae* from Lavan Island (eleven specimens); G2: *P. persiae* from Bandar Lengeh (one specimen); G3: *P. verruculata* from Bangka Island, Indonesia (one specimen). An interactive network is available by DOI (https://doi.org/10.18119/N9KW35).

For this purpose, the ethanolic extracts of *P. persiae* (clade 2) from the two Iranian localities of Lavan Island (G1: eleven specimens/combined) and of Bandar Lengeh (G2: one specimen) and one specimen of *P. verruculata* (clade 4) from Bangka Island, Indonesia (G3) were analyzed by HPLC coupled with high-resolution mass spectrometry and automated fragmentation (HPLC-HRMS/MS). The resulting MS/MS data were then subjected to the GNPS molecular networking platform. The resulting molecular network revealed 557 chemical features, visualized in nodes, and based on similarities in the fragmentation patterns of parent ion *m*/*z* signals were connected using edges (Fig. 4).

**Figure 5:**
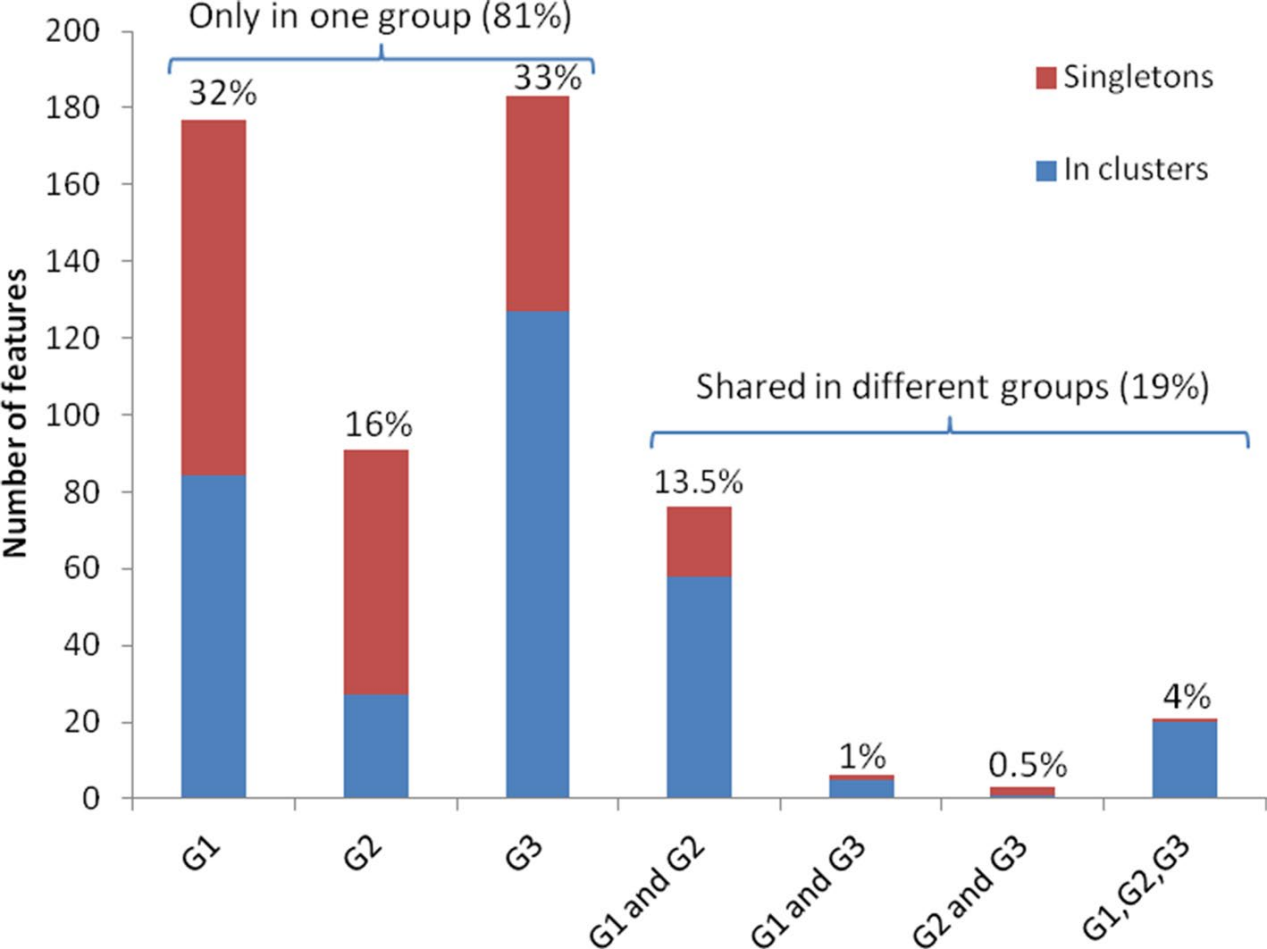
Contribution of the unique and shared chemical features in the molecular network. G1: *Peronia persiae* from Lavan Island (eleven specimens/combined); G2: *P. persiae* from Bandar Lengeh (one specimen); G3: *P. verruculata* from Bangka Island, Indonesia (one specimen).

Nodes were color-coded according to the three geographic groups. Among the 557 detected chemical features, 106 (19%) showed an overlap of at least two of the three groups. The biggest overlap was observed between *P. persiae* from Lavan Island and Bandar Lengeh (G1 and G2 with 76 nodes: 13.5%) and only 21 features (4%) were identified in the core metabolome of all three groups (Fig. 5). On the other hand, 451 nodes (81%) were detected only in one group, almost half them (213 nodes: 38%) were unique features with no similar MS spectra to other compounds (singletons) and another half (238 nodes: 43%) were connected by edges to other features or analogs in the same or other groups (cosine > 0.5).

In addition, a comparative analysis of the total UV absorption spectra (254 nm) of each sample (G1, G2, and G3) was pursued revealing nearly identical chromatograms of G1 and G2. Chromatogram G3, however, varied in peak counts, intensities, and retention times. In summary, several chemical features of the molecular network were putatively assigned based on their *m/z* ratio, UV absorption, and retention time using the GNPS library and the in silico dereplication tool Dereplicator-plus^22^ (Fig. 4, Table 1).

**Table 1:** List of chemical features putatively assigned using GNPS and dereplicator-plus from the resulting MS/MS data of *Peronia persiae*; Lavan Island, Iran (G1), *P. persiae;* Bandar Lengeh, Iran (G2), and *P. verruculata*; Bangka Island, Indonesia (G3).

Chemical feature	*m/z*	Retention time (min)	Detected group
Onchitriol I A	629.362 [M + H]^+^	15	G1, G2
Onchitriol I B	643.376 [M + H]^+^	15.8	G1, G2
Onchitriol I C	643.376 [M + H]^+^	13.8	G1, G2
Onchitriol I D	699.437 [M + H]^+^	18.6	G1, G2, G3
Onchitriol II B	601.368 [M + H]^+^	14.5	G1, G2
11,13-Dipropanoyl-Ilikonapyrone	657.391 [M + H]^+^	16.5	G1, G2, G3
11-(3-Methylbutanoyl)-13-propanoyl-Ilikonapyrone	685.418 [M + H]^+^	17.6	G1, G2, G3
Glycine betaine	257.147 [2 M + Na]^+^	1.1	G1, G2, G3
Proline betaine	283.164 [2 M + Na]^+^	1.2	G1, G2, G3
Hydroxyproline betaine	299.150 [2 M + Na]^+^	1.2	G3
Genistein	271.057 [M + H]^+^	9.2	G1
Daidzein	255.063 [M + H]^+^	7.8	G1
Kaempferol-3-O-galactoside	471.090 [M + Na]^+^	26.6	G2
Cholesterol	271.057 [M + H]^+^	12	G3
Phenylalanine conjugated chenodeoxycholic acids	1,043.710 [2 M + Na]^+^	16.1	G3

**Figure 6:**
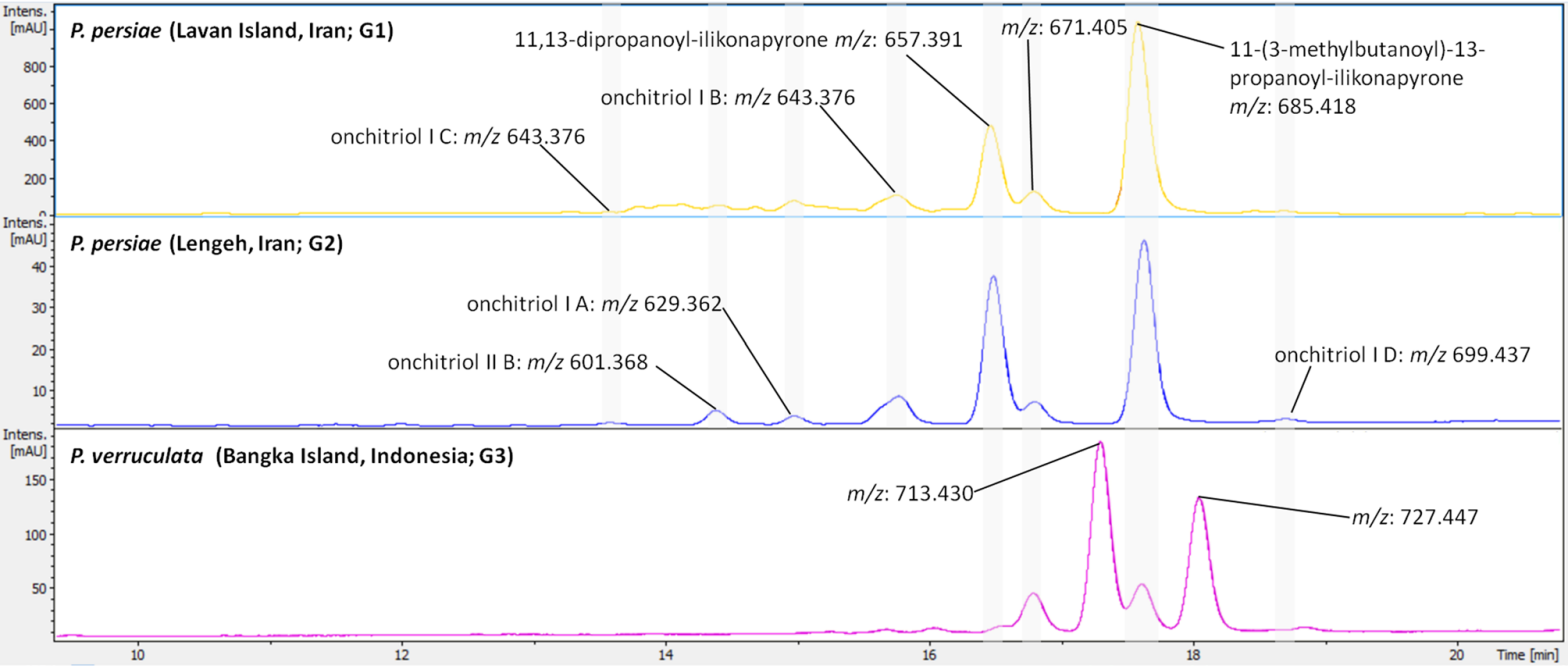
LC–UV (254 nm) trace of three groups of *Peronia* species extracts. Different chemical features assigned as polypropionate compounds are marked. Grey bars represent the same compounds found in different groups. MS/MS spectra are given in Figure S1.

**Figure 7:**
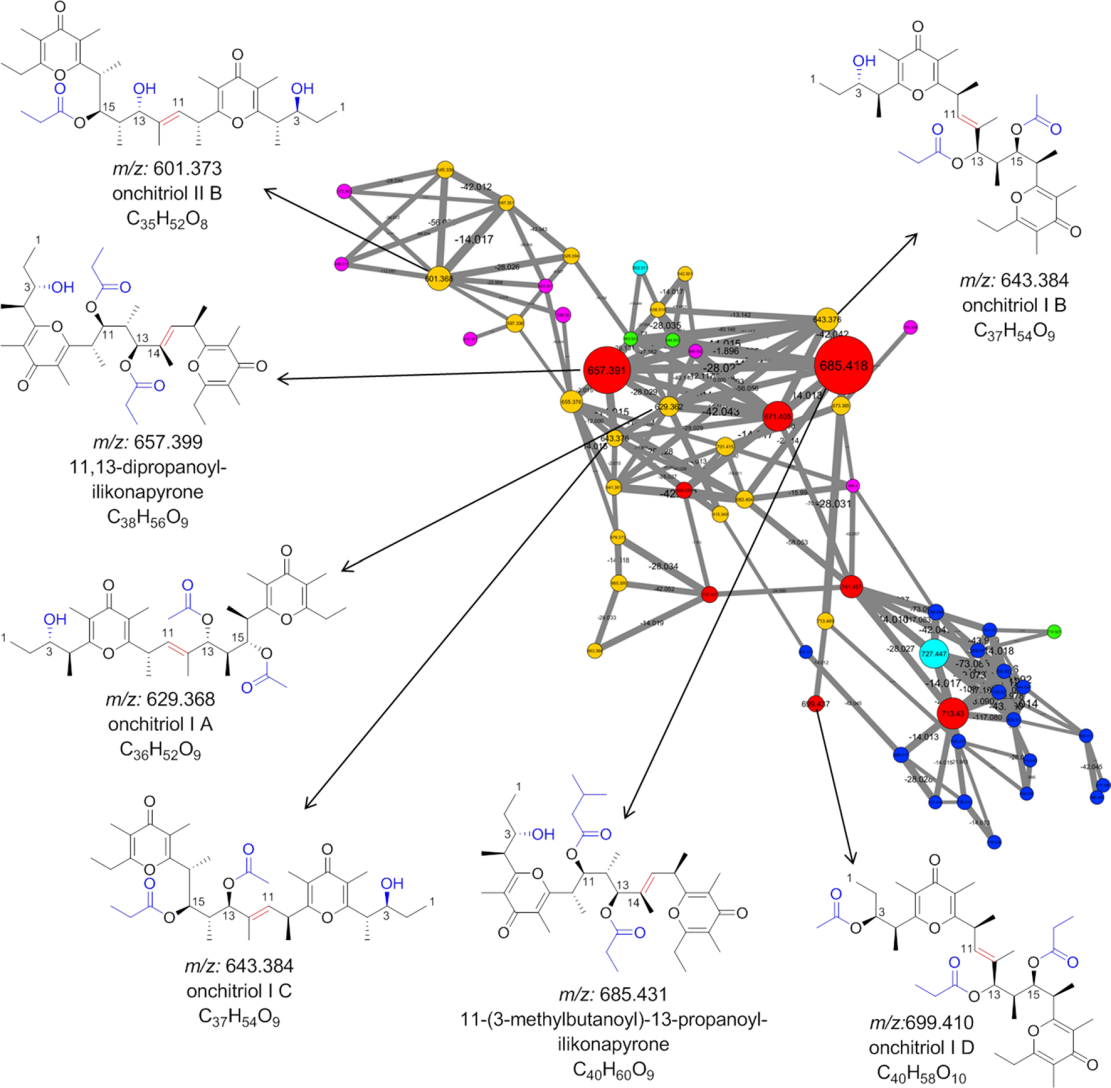
Dereplicated polypropionate esters in the molecular network of *Peronia persiae* and *P. verruculata* extracts. The backbone is in black and the different substitution of hydroxy, O-acetyl, O-propanoyl, and O-metylbutanoyl moieties are highlighted in blue. Indicative double bonds of onchitriols and ilikonapyrones are displayed in red. Node colors in subnetwork represent chemical features detected from different groups; pink (G1: *P. persiae* from Lavan Island, Iran), green (G2: *P. persiae* from Bandar Lengeh, Iran), dark blue (G3: *P. verruculata* from Bangka Island, Indonesia), orange (G1 and G2), light blue (G1/G2 and G3), and red (G1, G2, and G3).

As depicted in Fig. 4, the cluster containing most of the detectable chemical features was putatively assigned as polypropionate esters, which were also identified from other panpulmonates^17,18^. The largest nodes/chemical features within this cluster corresponded to the main UV peaks and highest signal in the LC–MS chromatograms of all groups (Figs. 6 and 7). Further analysis allowed for the tentative assignment of seven chemical features as onchitriol I A–D, onchitriol II B and two propanoyl-ilikonapyrones (Fig. 7). All derivatives share similar chemical features, such as 32 carbon atoms in their backbone with two γ-pyrone rings, but differ in the substitution of hydroxy, O-acetyl, O-propanoyl, and O-metylbutanoyl moieties on carbon 3, 13, 15 (onchitriols) or carbon 3, 11, 13 (ilikonapyrones). Onchitriols and ilikonapyrones also differ in the position of a double bond between C11–C12 or C14–C15, respectively (Fig. 7).

Comparison of G1, G2, and G3 showed that the relative ratio of these compounds differed between G1/G2 to G3. The main compounds assigned in G1 and G2 were onchitriol I B (*m/z*: 643.376 [M + H]^+^), 11,13-dipropanoyl-ilikonapyrone (*m/z*: 657.391 [M + H]^+^), an unknown derivative (*m/z*: 671.405), and 11-(3-methylbutanoyl)-13-propanoyl-ilikonapyrone (*m/z*: 685.418 [M + H]^+^), which all differ in 14 *m/z* units (Figs. 6 and S1). In contrast, the main compounds detected in G3 could not be assigned (*m/z*: 671.405, 713.430, 727.447) (Figs. 6 and S1) and relatively low intensities for 11-(3-methylbutanoyl)-13-propanoyl-ilikonapyrone (*m/z*: 685.418 [M + H]^+^) were detectable.

**Figure 8:**
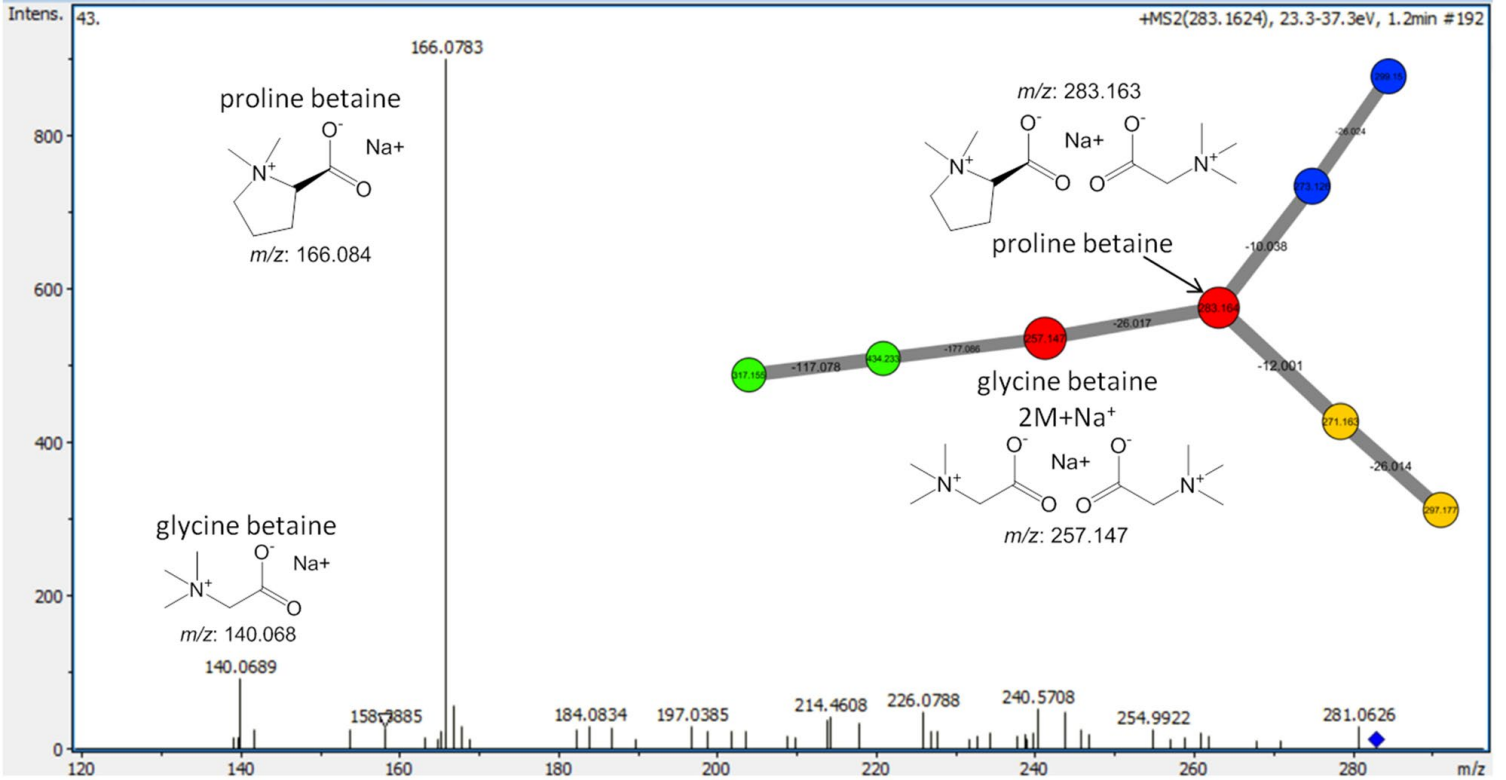
Amino acid-betaine cluster dereplicated from extracts of both *Peronia persiae* and *P. verruculata* with different localities. MS/MS spectra of proline betaine complex are given here and the MS/MS spectra of glycine betaine and hydroxyproline betaine are given in the supplementary data (Figure S2). Node's colors represent chemical features detected from different groups; green (G2: *P. persiae* from Bandar Lengeh, Iran), blue (G3: *P. verruculata* from Bangka Island, Indonesia), orange (G1: *P. persiae* from Lavan Island, Iran and G2), and red (G1, G2, and G3).

Another characteristic cluster, assigned by GNPS and found in all three groups contained very hydrophilic compounds (RT: 1.1–1.2 min) belonging to the class of amino acid-betaine compounds. Glycine betaine was detected as *m/z*: 257.147 [2 M + Na]^+^ which is connected to another node with the *m/z* shift of 26.017. This corresponds to the mass of proline betaine zwitterion complex, together with glycine betaine having an *m/z* of 283.164 (Fig. 8). Compounds of this class are zwitterions and contain a quaternary ammonium cation and a carboxylate anion. The MS/MS fragmentation of both complexes revealed the monomeric ions with corresponding masses of sodium adducts (Fig. 8). Another node in this cluster corresponds to hydroxyproline betaine (Figure S2).

**Figure 9:**
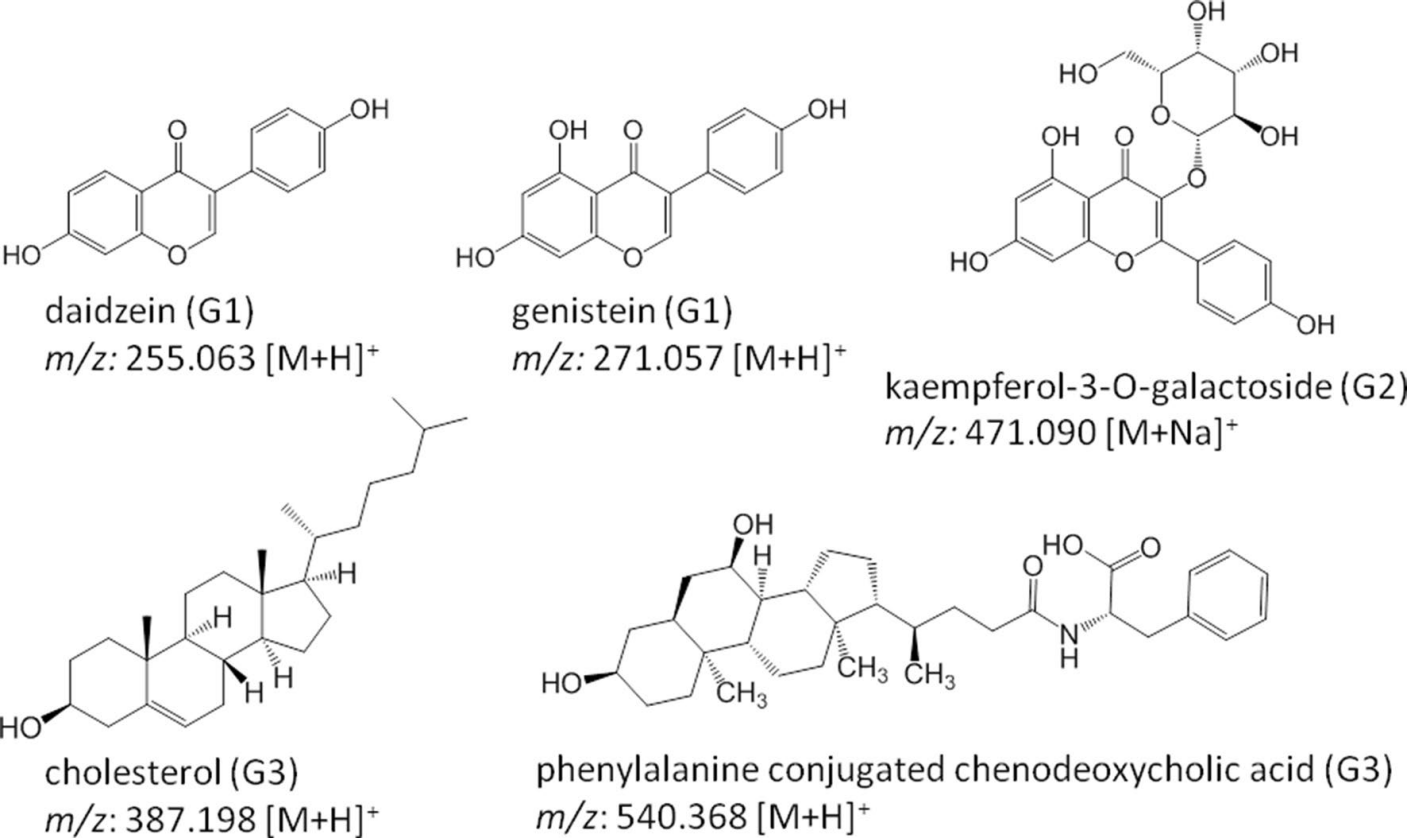
Unique features dereplicated only in one of the investigated *Peronia* species. G1: *P. persiae* from Lavan Island, Iran, G2: *P. persiae* from Bandar Lengeh, Iran, and G3: *P. verruculata* from Bangka Island, Indonesia. Mirror MS/MS spectra against GNPS library spectra are given in Figures S3 and S4.

Furthermore, flavonoid compounds typically produced by plants^23^, such as genistein (*m/z*: 271.057 [M + H]^+^) and daidzein (*m/z*: 255.063 [M + H]^+^), were detected solely within G1 and G2, while the glycosylated compound kaempferol-3-O-galactoside was exclusively detected in G2 (Figs. 9 and S3).

The second species-specific cluster found only in *P. verruculata* (G3) was annotated as cholesterol and conjugated chenodeoxycholic acids (Figs. 9 and S4).

## Discussion

Despite many systematic and phylogenetic analyses of Onchidiidae^9,24,25^, our knowledge of the species diversity and distribution, especially in the genus *Peronia,* is still rudimentary. The ABGD test of the COI gene of genus *Peronia* revealed nine well supported distinct clades and the constructed haplotype network confirms the ABGD test result. This is in line with divergence distance results in our previous study^3^ and demonstrates that haplotype networking can be useful for the delimitation of putative cryptic species, even if anatomical studies are not available. The level of mutations in each clade with more than two specimens (clades 1, 2, and 4) was relatively low, whereas the distances between the clades are high. With our analysis here, we could confirm the distinctiveness of *P. persiae* (clade 2) from all other clades by haplotype network analyses. Our metabolomic investigations also indicate its chemical distinctiveness from the second *Peronia* species investigated here, *P. verruculata* (clade 4).

In contrast to all other clades, clade 4 (*P. verruculata*) shows a very wide distribution from Oman to southern China and even Australia. Many unassigned specimens in this clade were collected from Singapore. Interestingly, clade 1 comprises only sequences from this particular locality. Chang et al. reported that the specimens of *Peronia* sp. from Singapore are morphologically very similar^6^, while our results on genetic distance analysis clearly separate the Singapore specimens into two groups. Clade 1 is an undescribed cryptic species, whereas clade 4 includes other haplotypes, partly identified as *P. verruculata*. *Peronia verruculata* from Bandra in India was described morphologically in great detail by Awati and Karandikar^2^. All available *P. verruculata* haplotypes used in our analysis have not been described morphologically. Therefore, the assignment of all other unnamed *Peronia* haplotypes within the clade 4, including our sequence from Indonesia, to *P. verruculata* should be regarded as preliminary until a confirmed reference sequence of this species is available.

The distribution of the intertidal fauna and flora are greatly affected by hydrographical conditions^26^. This certainly also holds for members of the intertidal living Onchidiidae. The habitat shifts and changes in abiotic factors such as salinity, temperature, oxygen level, and light intensity towards higher extremes have affected the evolution and radiation of the panpulmonate molluscs^27–29^. Our result on the chemical composition of *P. persiae* from different localities in the Gulf (Lavan Island vs. Bandar Lengeh) that generally encounter higher temperature and salinity values^3^ showed a similar mixture of the main chemical features in their body extracts, thus indicating no regional differences within this species. However, the chemical composition was different from the *P. verruculata* specimen from Indonesia.

Common secondary metabolites detected within all *P. persiae* specimens were the flavonoids genistein and daidzein, with the glycosylated form only found in the single specimen from Bandar Lengeh. These compounds are widely distributed in plants and are known to be potent antioxidants. Therefore, these metabolites may help slugs in the intertidal zone with a high level of oxidative stress induced by desiccation during low tides^30,31^. Indeed, genistein showed antimutagenic effects with DNA damage agents at a concentration of 10 μM^32^. A similar bioactive flavonoid, apigenin, was also isolated from the intertidal aplysiid heterobranch *Syphonota geographica* (A. Adams and Reeve, 1850) (Aplysiidae, Tectipleura) and its food, the seagrass *Halophila stipulacea* (Forsskål) Ascherson, 1867^33^.

These findings are indicative for the food dependency of *P. persiae* for these flavonoids as members of this genus are found grazing on intertidal algae-covered rocks^1^. Secondary metabolites of dietary origin are known to be modified by the sea slugs, providing them a wider range of ecological opportunities^33^. Here it could be speculated that *P. persiae* either feeds and/or accumulates plant-derived metabolites for defensive purpose granting them a better survival probability.

In contrast, cholesterol and bile acids were exclusively identified from *P. verruculata.* Bile acids are water-soluble steroids formed during the catabolism of cholesterol to primary bile acids, i.e. cholic acids and chenodeoxycholic acids^34^ and show anticancer and antibacterial activities^35,36^. Cholesterol was previously reported from *Onchidium reevesii* (Gray, 1850)^17^, octocorals^37^, and sponges^38^, while there are only a few reports of bile acids and derivatives in other marine invertebrates. Bile acid derivatives in marine invertebrates are considered to be of symbiotic microbial origin^39^. In this case, *P. verruculata* should have a symbiotic bacterial strain that produces the bile acids which then can be conjugated with different amino acids to provide chemical defense for the host, an interesting hypothesis that has to be proven yet.

Compounds that were found in both species also included a diverse mixture of polypropionate esters as the most common and prominent chemical features. Polypropionates are reported from various Onchidiidae, including *Onchidium reevesii*^17^, partly described under the former name *Onchidium struma* (nomen nudum)^40^, various unidentified *Onchidium* species^18,19,41^, and also *P. verruculata*^42^. Polypropionates with different carbon skeleton were also described from genus *Siphonaria* (Gastropoda: Tectipleura) such as *Siphonaria baconi* Reeve, 1856 (now accepted as *Siphonaria zelandica* Quoy and Gaimard, 1833)^43^, and *Siphonaria diemenensis* Quoy and Gaimard, 1833^44^. Moreover, other marine molluscs, like photosynthetic members of the order Sacoglossa, harbor a big complex of different polypropionates acting as photoprotectants and antioxidants within the slugs^45,46^. These compounds seem to be common within the Onchidiidae and are reported from various localities, including China, Hawaii, and Australia^18,44,47^. However, the diversity and contribution of these metabolites with respect to each species was not reported before. Onchitriol and Ilikonapyrone compounds are polypropionates whose skeletons contain 32 carbon atoms, two γ-pyrone rings, and several hydroxy groups^19^. They were previously isolated from *P. verruculata* (as *Onchidium verruculatum*)^42^, and also from other members assigned to the genus *Onchidium* (as *Onchidium* sp., *Onchidium* sp.1, and *Onchidium* sp.2)^18,41^. The identity of these species, even genus affiliation, has not yet been fully verified and the assignment should be considered with caution.

Onchitriol and Ilikonapyrone compounds were detected during our study in *P. persiae* and *P. verruculata,* although, the two species showed different ratios and types of these polypropionate compounds in their body extracts. Other polypropionate compounds such as onchidiol^48,49^, onchidionol^49^ and onchidione^49,50^ from *Onchidium* sp., and peroniatriols from *P. peronii*^51^ could not be dereplicated from the here investigated species. A common polyketide biosynthetic origin in panpulmonates is proposed for polypropionate metabolites, and the diversity of these compounds can arise when the respective polyketide chain precursors folded in a different manner^44,47^. These metabolites are shown to repel predators such as shrimps, with a minimum effective dose of 1.0 mg/mL^50^. Therefore, the major presence of these lipophilic metabolites in *Peronia*, and also other onchidiid species suggests their involvement in the chemical defense of the slugs as deterrence agents against predators. Additionally, interesting antitumor, anticancer, as well as antiviral activities have been shown from some of these polypropionate compounds^18,19,52^.

An additional important compounds cluster detected in both *P. persiae* and *P. verruculata* has been assigned to the hydrophilic zwitterionic amino acid-betaine compounds. They are major osmolytes in response to hyperosmotic conditions that accumulate in different concentrations during the stress time. Therefore, they have a critical role during the low tide in the presence of evaporation and increased salinity. Indeed, proline betaine was reported as an effective osmolyte in the extremely euryhaline sea slug, *Elysia chlorotica* Gould, 1870 (Sacoglossa, Gastropoda)^53^.

To the best of our knowledge, the dereplicated flavonoids, bile acids, and amino acid-betaine compounds were not reported from *Onchidiidae* before. However, similar flavonoids from *Syphonota geographica*^33^, betaines from *Elysia chlorotica*^53^, and bile acid derivatives from octocorals^37^ and sponges^38^ were previously reported from other sea slugs or marine invertebrates.

To conclude, our investigation of the genus *Peronia* revealed nine distinct clades of COI gene sequences, with *P. persiae* forming a chemically distinct species, only distributed in the Persian Gulf. All other clades, except *P. verruculata*, showed a geographically narrow distribution. Molecular networking revealed a large chemical diversity including compounds, which have not been reported from Onchidiidae, or even panpulmonata before. Species or geographically related compounds are antioxidant flavonoids, found only in *P. persiae,* and cholesterol and its derived conjugated chenodeoxycholic acids in *P. verruculata*. A major common group of food deterrent compounds shared by both species comprises polypropionates, which differed only in type and ratio between *P. persiae* and *P. verruculata*, as well as the osmoprotectant amino acid-betaine compounds important to cope with osmotic stress. In how far all the other clades are characteristic in their chemical composition and the value of chemotaxonomy remains to be investigated for all other cryptic and undescribed species, as well as those *Peronia* species, for which no molecular barcodes are available at the moment.

## Materials and methods

*Peronia persiae* specimens were collected in the intertidal zone during low tide from the surface of rock or rock crevices at Bandar Lengeh, Iran (26° 33′ 29″ N 54° 52′ 50″ E) in March 2015 (one specimen), and Lavan Island, Iran (26° 48′ 20.99″ N 53° 16′ 4.80″ E) in February 2016 (eleven specimens). One specimen of *P. verruculata* (assigned as *Peronia* sp. a in Papu et al. 2020 and as *Peronia* sp. 7 in Maniei et al. 2020) was collected at Bangka Island, Indonesia (2° 15′ 0″ S, 106° 0′ 0″ E) in September 2018 (Table S1). All specimens were 4–6 cm in length and preserved in EtOH 96%. Small pieces of the foot were used for molecular barcoding, and the preservation alcohol was used for metabolomic experiments. Sequenced specimens of *P. persiae* are deposited as a voucher at the Zoologische Staatssammlung München, Germany. The specimen *P. verruculata* is part of the reference collection of Sam Ratulangi University, Manado, Indonesia and was kindly provided by A. Papu.

### DNA extraction, PCR, and DNA sequencing

DNA isolation was carried out using the Qiagen DNeasy Blood and Tissue kit, following the manufacturer’s instructions. Partial sequences of mitochondrial COI (ca. 680 bp) were amplified by polymerase chain reaction (PCR) using the primers LCOI490-JJ (5′-CHACWAAYCATAAAGATATYGG-3′) and HCO2198-JJ (5′-AWACTTCVGGRTGVCCAAARAATCA-3′)^54^ for COI,16Sar-L (5′-CGCCTGTTTATCAAAAACAT-3′). The following thermoprofile during the PCR was used: 15 min at 95 °C; 40 cycles with following reaction conditions were involved: initial denaturation at 94 °C for 35 s, subsequent annealing at 55 °C for 90 s, elongation at 72 °C for 90 s and final elongation step of 72 °C for 10 min. Sequencing was performed by Macrogen Europe (Amsterdam, Netherlands). Sequences are deposited in GenBank with the accession numbers listed in Table S2.

### Phylogenetic reconstruction

*Peronia* sequences were downloaded from NCBI, and identical sequences removed. The final alignment contained 157 sequences including eleven sequences of *P. persiae* from both localities and the one sequence of *P. verruculata* from Bangka Island (see Table S2) with two sequences of *Wallaconchis graniferus* as outgroup.

Sequences were edited using BioEdit (ver.7.2.6.1)^55^ and aligned using MAFFT^56^ in Geneious v7.1.9^57^. The maximum likelihood (ML) analysis was performed in IQ-TREE^58,59^, using the online version 1.6.3 on a web server (https://iqtree.cibiv.univie.ac.at/). The evolutionary model GTR was applied. Support values were calculated based on 1,000 ultrafast bootstrap replicates and the approximate likelihood ratio test (SH-aLRT) (2,000 replicates) was applied. Dendroscope (version 3.5.8)^60^ and Inkscape (version 0.92) (https://inkscape.org/en/) were used to edit the phylogram.

*Species delimitation* The Automatic Barcode Gap Discovery test (ABGD)^61^ was applied for delimiting the species within this CO1 data set using default values and the evolutionary model Kimura K80. This test is independent of predefined species groups^61,62^.

### Haplotype networking

A statistical parsimony analysis^63^ was conducted with all individual COI sequences from the 157 CO1 sequences containing data set already used for the phylogenetic reconstruction and species delimitation test, using the program TCS v.1.21^64^ in PopART ^65^ with a 95% connection limit and 5,000 iterations. This program helps to identify haplotypes that were shared among individuals, and also calculates the number of substitutions connecting haplotypes in the network (Templeton et al.^63^. The program also allows for including and visualizing geographic information within the network.

### Metabolite extraction and HPLC–MS/MS analysis

The preservation alcohol of all eleven *P. persiae* specimens from Lavan Island, Iran was combined and subsequently analyzed for chemical composition (Group 1 or G1). The preservation alcohol of the single specimen of *P. persiae* from Bandar Lengeh, Iran was analyzed separately (G2), as was the one specimen of *P. verruculata* from Bangka Island, Indonesia (G3). The EtOH of each group was evaporated under vacuum conditions, the residue was re-dissolved in 100 µL methanol and analyzed by a micrOTOF-QIII mass spectrometer (Bruker) with ESI-source coupled with an HPLC Dionex Ultimate 3000 (Thermo Scientific) using an EC10/2 Nucleoshell C18 2.7 µm column (Macherey–Nagel). The column temperature was 25 °C. MS data were acquired over a range from 100–3,000 m*/z* in positive mode. Auto MS/MS fragmentation was achieved with rising collision energy (35–50 keV over a gradient from 500–2000 *m/z*) with a frequency of 4 Hz for all ions over a threshold of 100. HPLC begins with 90% H_2_O containing 0.1% acetic acid. The gradient starts after 1 min to 100% acetonitrile (0.1% acetic acid) in 20 min. 5 µl of a 1 mg/ml sample solution (MeOH) was injected into a flow of 0.3 ml/min^66^.

### Molecular networking

All MS/MS data of each group (G1, G2, and G3) were converted to mzXML format and transferred to the Global Natural Product Social Molecular Networking (GNPS) server (gnps.ucsd.edu)^21^ and the molecular network was created by the online workflow at GNPS^21^ using the spectra with a minimum of four fragment ions and by merging all identical spectra into nodes, representing parent masses. Compounds with similar fragmentation patterns are connected by edges, displaying molecular families with similar structural features. The data were filtered by removing all MS/MS peaks within + /− 17 Da of the precursor *m/z*. MS/MS spectra were window filtered by choosing only the top 6 peaks in the + /− 50 Da window throughout the spectrum. The resulting data were then clustered by MS-Cluster with a parent mass tolerance of 0.02 Da and an MS/MS fragment ion tolerance of 0.02 Da to create consensus spectra. Further, consensus spectra that contained less than 2 spectra were discarded. A network was then created where edges were filtered to have a cosine score above 0.5 and more than 4 matched peaks. Further edges between two nodes were kept into the network if and only if each of the nodes appeared in each other's respective top 10 most similar nodes. The spectra in the network were then searched against GNPS spectral libraries. The library spectra were filtered in the same manner as the input data including analog search. All matches kept between network spectra and library spectra were required to have a score above 0.5 and at least four matched peaks. Furthermore, DEREPLICATOR plus was used for in silico identification of both peptidic and non-peptidic natural products^22^. The network was visualized via Cytoscape 3.6.1. The molecular network file is available at the NDEx site^67^ (https://doi.org/10.18119/N9KW35).

## Supplementary information

Supplementary Information.
