# Supplementary material for: From Persian Gulf to Indonesia: interrelated phylogeographic distance and chemistry within the genus Peronia (Onchidiidae, Gastropoda, Mollusca)
Source: Sci Rep. 2020 Aug 3;10:13048. doi: 10.1038/s41598-020-69996-8 (PMC7400755; doi:10.1038/s41598-020-69996-8)
Supplement: Supplementary file 1 — Supplementary Information. [file 41598_2020_69996_MOESM1_ESM.docx]

**Supplementary information**

**From Persian Gulf to Indonesia: Interrelated phylogeographic distance and chemistry within the genus *Peronia* (Onchidiidae, Gastropoda, Mollusca)**

Fatemeh Maniei^1^, Jamshid Amiri Moghaddam^2,*^, Max Crüsemann^3^, Christine Beemelmanns^2^, Gabriele M. König^3^, Heike Wägele^1^

*^1^Zoologisches Forschungsmuseum Alexander Koenig, Bonn, Germany*

*^2^ Leibniz Institute for Natural Product Research and Infection Biology e.V. Hans-Knöll-Institute (HKI), Jena, Germany*

*^3^Institute for Pharmaceutical Biology, Bonn, Germany*

^*^Corresponding author. Email: Jamshid.Amiri-Moghaddam@hki-jena.de
Running title: Phylogeography and chemistry of *Peronia*

**Table S1.** Specimens used in this study. Abbreviations of locality: BL = Bandar Lengeh (Iran); LA = Lavan Island (Iran); BA = Bangka Island (Indonesia)

| **Specimen** | **Preservation** | **Purpose** | **Length of preserved animal (mm)** |
| --- | --- | --- | --- |
| BL 1, *P. persiae* | ETOH 96% | Haplotype network, chemistry | 37 |
| LA 1, *P. persiae* | ETOH 96% | Haplotype network, chemistry | 32 |
| LA 2, *P. persiae* | ETOH 96% | Haplotype network, chemistry | 22 |
| LA 3, *P. persiae* | ETOH 96% | Haplotype network, chemistry | 26 |
| LA 4, *P. persiae* | ETOH 96% | Haplotype network, chemistry | 28 |
| LA 5, *P. persiae* | ETOH 96% | Haplotype network, chemistry | 34 |
| LA 6, *P. persiae* | ETOH 96% | Haplotype network, chemistry | 32 |
| LA 7, *P. persiae* | ETOH 96% | Haplotype network, chemistry | 35 |
| LA 8, *P. persiae* | ETOH 96% | Haplotype network, chemistry | 25 |
| LA 9, *P. persiae* | ETOH 96% | Haplotype network, chemistry | 22 |
| LA 10  *P. persiae* | ETOH 96% | Haplotype network, chemistry | 13 |
| LA 12  *P. persiae* | ETOH 96% | Chemistry | 31 |
| BA  *P.* *verruculata*  (*Peronia* sp. a in Papu et al. 2020/ *Peronia* sp7 in Maniei et al. 2020) | ETOH 96% | Haplotype network, chemistry | 25 |

**Table S2.** Specimens sequenced for this study and sequences obtained from GenBank, including locality, GenBank accession numbers, and geographical coordination.

| **Confirmed name of Species** | **Locality** | **GenBank COI** | **Coordinate/**  **Longitude** | **Coordinate/**  **Latitude** |
| --- | --- | --- | --- | --- |
| *Peronia* sp. | Philippines | HQ660050 | 122.96777353 | 9.785934 |
| *Peronia verruculata* | Australia, Queensland | EF489391 | 142.702796 | -20.917574 |
| *Peronia* sp. 1 | Hawaii | HQ660038 | -155.582782 | 19.896766 |
| *Peronia* sp. 2 | Oman | HQ660044 | 55.975413 | 21.473533 |
| *Peronia* sp. 3 | Australia, Queensland | HQ660048 | 142.702796 | -20.917574 |
| *Peronia* sp. 4 | Mozambique | HQ660045 | 34.187957 | -24.916742 |
| *Peronia* sp. 5 | Mozambique | HQ660047 | 34.187957 | -24.916742 |
| *Peronia* sp. 6 | Indonesia, Sulawesi | HQ660046 | 120.5279 | -1.8479 |
| *Peronia* sp. | China, Hainan. | HQ285979 | 110.349229 | 20.017378 |
| *Peronia* sp. | China, Hainan | HQ285980 | 110.349229 | 20.017378 |
| *Peronia* sp. | China, Hainan | HQ285981 | 110.349229 | 20.017378 |
| *Peronia ‎* sp. | China, Hainan. | JN543165 | 110.349229 | 20.017378 |
| *Peronia* sp. | Japan, Okinawa | HQ660043 | 127.680932 | 26.212401 |
| *Peronia peronii* | Guam | HQ660041 | 144.766159 | 13.433168 |
| *Peronia cf. peronii* | Mozambique | HQ660042 | 34.187957 | -24.916742 |
| *Peronia verruculata* | China, Fujian | GU166566 | 120.216978 | 27.324479 |
| *Peronia ‎ verruculata* | China, Fujian | GU166564 | 120.216978 | 27.324479 |
| *Peronia ‎ verruculata* | China, Fujian | GU166563 | 120.216978 | 27.324479 |
| *Peronia ‎ verruculata* | China, Hainan | GU166561 | 110.349229 | 20.017378 |
| *Peronia ‎ verruculata* | China, Hainan | GU166559 | 110.349229 | 20.017378 |
| *Peronia ‎ verruculata* | China, Hainan | GU166558 | 110.349229 | 20.017378 |
| *Peronia ‎ verruculata* | China, Hainan | GU166562 | 110.349229 | 20.017378 |
| *Peronia ‎ verruculata* | China, Hainan | GU166560 | 110.349229 | 20.017378 |
| *Peronia ‎ verruculata* | China, Fujian | GU166565 | 120.216978 | 27.324479 |
| *Peronia ‎ verruculata* | China, Hainan | GU166557 | 110.349229 | 20.017378 |
| *Peronia ‎ verruculata* | China, Fujian | JN543154 | 120.216978 | 27.324479 |
| *Peronia ‎ verruculata* | China, Hainan | JN543153 | 110.349229 | 20.017378 |
| *Peronia ‎ verruculata* | China, Zhanjiang | JN543152 | 110.359368 | 21.270702 |
| *Peronia persiae* | Iran, Bandar lengeh | MK312167 | 54.888679 | 26.562787 |
|  | Iran, Lavan Island | MK993386-  MK993395 | 53.268 | 26.805831 |
| *Peronia verruculata* (*Peronia* sp. a in Papu et al. 2020/ *Peronia* sp7 in Maniei et al. 2020) | Indonesia, Bangka | MK993397 | 125.153055 | 1.79666666 |
| *Peronia* sp. | Singapore | MH002607 | 103.851959 | 1.290270 |
|  |  | MH002590 |  |  |
|  |  | MH002605 |  |  |
|  |  | MH002603 |  |  |
|  |  | MH002600 |  |  |
|  |  | MH002599 |  |  |
|  |  | MH002594 |  |  |
|  |  | MH002591 |  |  |
|  |  | MH002589 |  |  |
|  |  | MH002586 |  |  |
|  |  | MH002585 |  |  |
|  |  | MH002580-MH002582 |  |  |
|  |  | MH002575-MH002578 |  |  |
|  |  | MH002592 |  |  |
|  |  | MH002596 |  |  |
|  |  | MK142731 |  |  |
|  |  | MK142730 |  |  |
|  |  | MK142725-MK142728 |  |  |
|  |  | MK142720- MK142723 |  |  |
|  |  | MK142717 |  |  |
|  |  | MK142715 |  |  |
|  |  | MK142714 |  |  |
|  |  | MK142712 |  |  |
|  |  | MK142704 |  |  |
|  |  | MK142706-MK142709 |  |  |
|  |  | MH002597 |  |  |
|  |  | MH002604 |  |  |
|  |  | MH002579 |  |  |
|  |  | MH002598 |  |  |
|  |  | MH002602 |  |  |
|  |  | MH002583 |  |  |
|  |  | MK142710 |  |  |
|  |  | MH002593 |  |  |
|  |  | MK142729 |  |  |
|  |  | MK142713 |  |  |
|  |  | MH002595 |  |  |
|  |  | MK142732 |  |  |
|  |  | MK142724 |  |  |
|  |  | MK142716 |  |  |
|  |  | MH002588 |  |  |
|  |  | MK142705 |  |  |
|  |  | MK142719 |  |  |
|  |  | MK142711 |  |  |
|  |  | MH002584 |  |  |
|  |  | MH002606 |  |  |
|  |  | MH002601 |  |  |
|  |  | MK142718 |  |  |
| *Peronia* sp. 2 | Singapore | MK142680-MK142703 | 103.851959 | 1.290270 |
|  |  | MH002574 |  |  |
|  |  | MH002573 |  |  |
|  |  | MH002569 |  |  |
|  |  | MH002567 |  |  |
|  |  | MH002566 |  |  |
|  |  | MH002559-MH002563 |  |  |
|  |  | MH002548-MH002556 |  |  |
|  |  | MH002546 |  |  |
|  |  | MH002545 |  |  |
|  |  | MH002571 |  |  |
|  |  | MH002572 |  |  |
|  |  | MH002547 |  |  |
|  |  | MK142694 |  |  |
|  |  | MK142689 |  |  |
|  |  | MH002564 |  |  |
|  |  | MH002550 |  |  |
|  |  | MH002557-MH002558 |  |  |
|  |  | MH002570 |  |  |


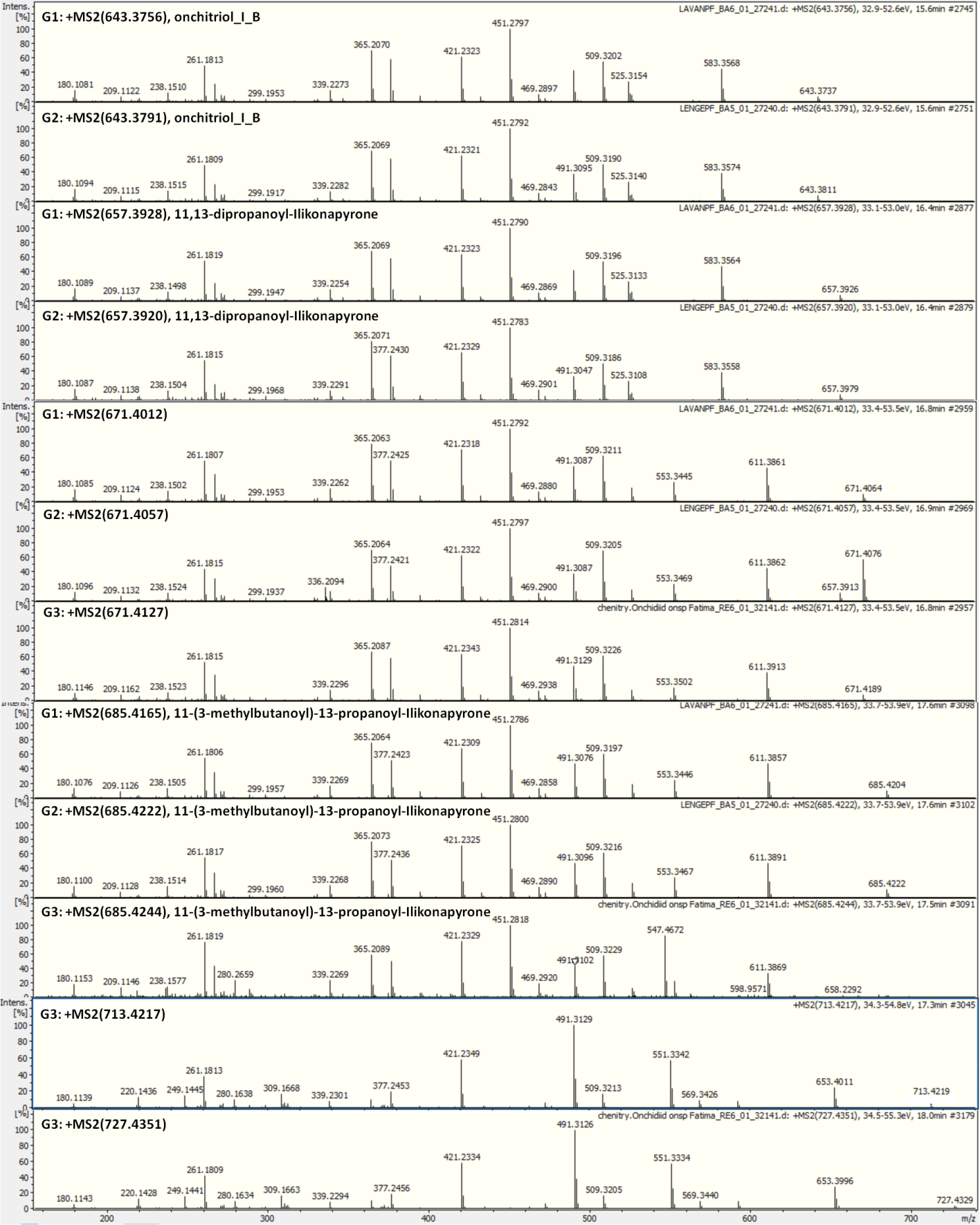
**Figure S1.** MS/MS spectra of the main polypropionate compounds in three groups of *Peronia* extracts. G1: *P. persiae* (Lavan Island, Iran), G2: *P. persiae* (Bandar Lengeh, Iran), and G3: *P.* *verruculata* (Bangka Island, Indonesia).


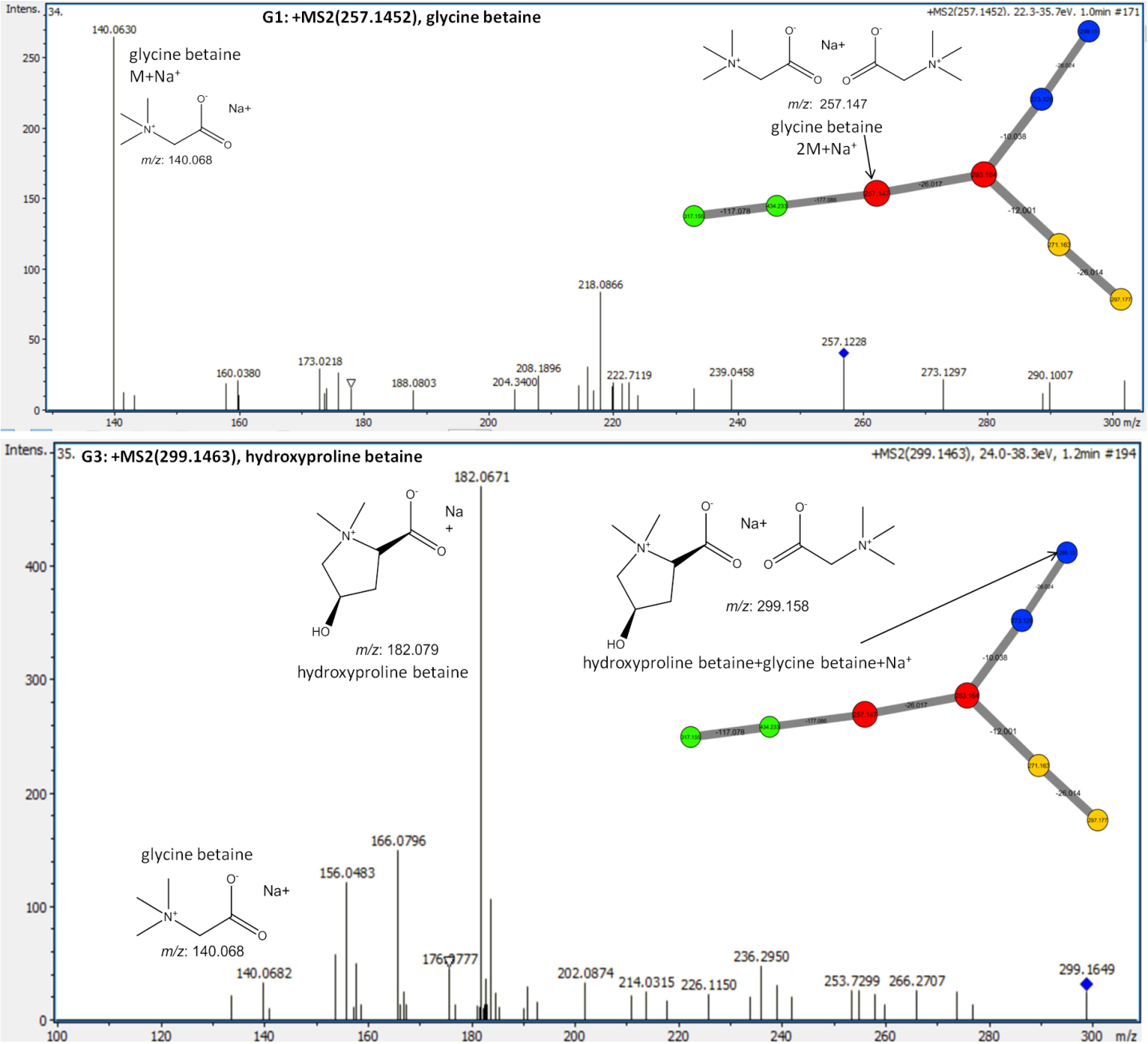
 **Figure S2.** MS/MS spectra of glycine betaine in *Peronia persiae* and hydroxyproline betaine in *P.* *verruculata*.


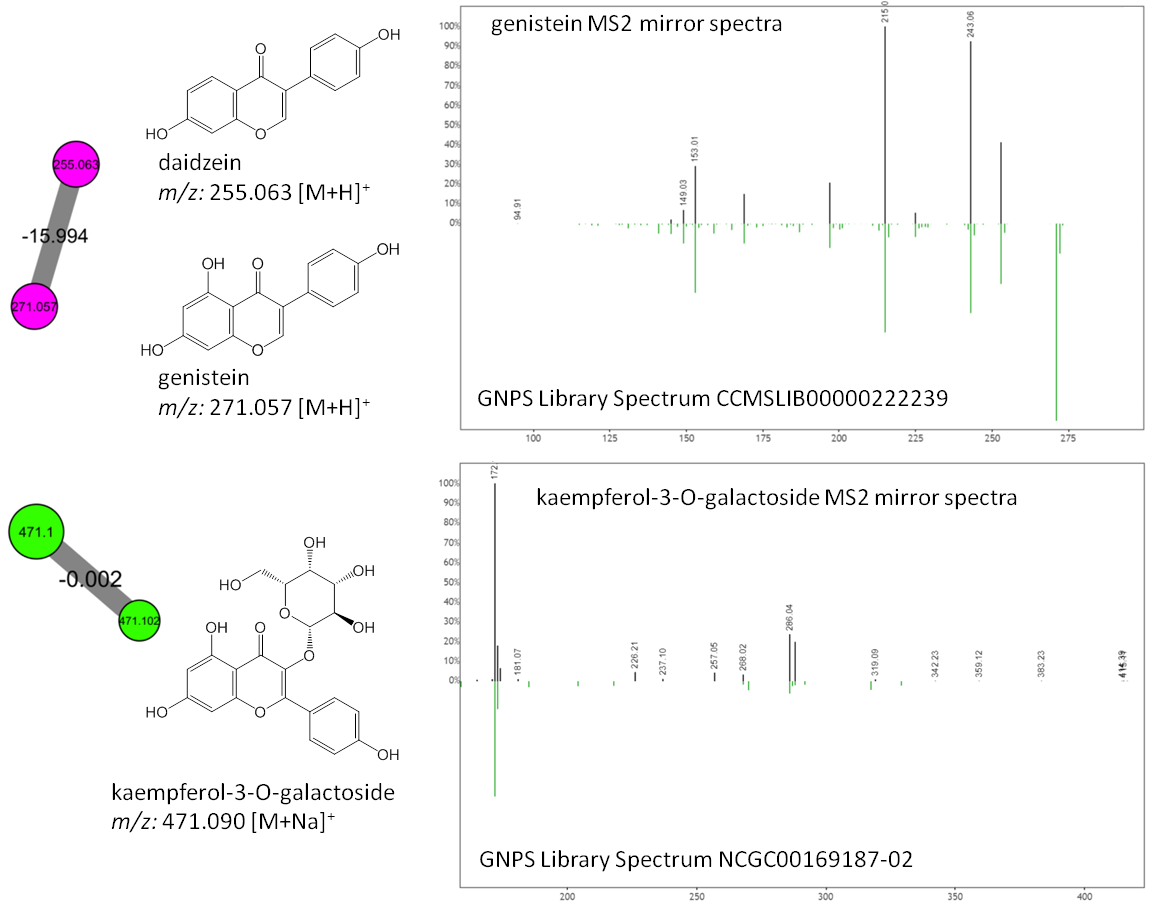
 **Figure S3.** Flavonoid compounds dereplicated from *Peronia persiae* from different localities.


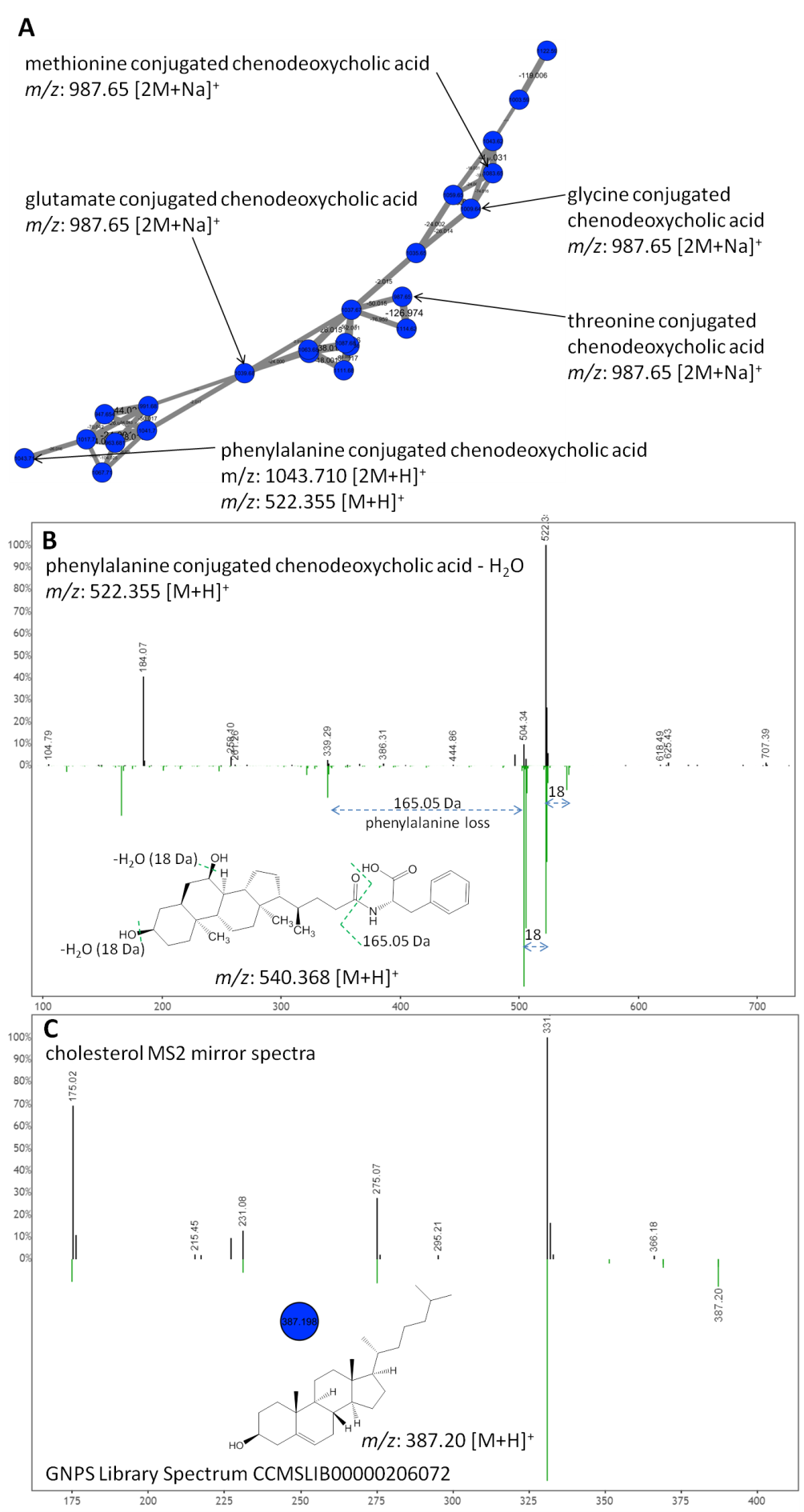
 **Figure S4.** Dereplicated conjugated chenodeoxycholic acid cluster and cholesterol found in *Peronia* *verruculata*.
